# Admission patterns and risk factors linked with neonatal mortality: A hospital-based retrospective study

**DOI:** 10.12669/pjms.36.6.2281

**Published:** 2020

**Authors:** Mohammed Mahmoud Al-Momani

**Affiliations:** 1 Mohammed Mahmoud Al-Momani, PhD, RN. Department of Community Health Sciences, College of Applied Medical Sciences, King Saud University, Kingdom of Saudi Arabia

**Keywords:** Neonates, Mortality, Apgar score, Neonatal Sepsis

## Abstract

**Objective:**

This research was conducted to analyze the elements and factors that link with newborn deaths at Neonatal Intensive Care Unit (NICU) of a public teaching hospital in Jordan.

**Methods:**

A retrospective study was conducted by reviewing the medical records of all the neonates admitted to the NICU over a one- year period from 1^st^ of March, 2018 to 28^th^ of February, 2019 at Princess Rahma Pediatric Hospital, Jordan.

**Results:**

Overall, the medical records of 1247 neonates were included in this study. The common causes of admission were sepsis (27.3%), followed by respiratory distress syndrome (RDS) (24.9%) and asphyxia (13.1%). The majority of the admitted neonates survived (91.9%), and the overall mortality rate was 8.1%. According to the cause-specific death rate analysis, RDS was the most common cause of death rate (35.6%), followed by sepsis (27.7%). Logistic regression analysis results show that gestational age, weight of the baby at birth, and the quick clinical assessment (Apgar) within the first five minutes were the strongest predictors of neonatal mortality (P<0.05).

**Conclusion:**

Sepsis, RDS, and asphyxia were the leading causes of morbidity in neonates. These diseases were also responsible for a high rate of mortality. Understanding the cause of morbidity and mortality of neonates admitted at NICU is useful for prioritizing and planning health services, re-allocating resources, and improving the quality of care.

## INTRODUCTION

The neonatal phase, which is the first 28 days of an infant’s life, is a vulnerable period due to many reversible severe illnesses. Low infant birth weight and premature babies are at a higher risk and are universally acknowledged as the ranking causes of morbidity and neonatal deaths.^1^

According to the World Health Organization, of the 130 million newborns, four million will die during the neonatal period, and half neonatal deaths (i.e., 50%) occur within the first 24 hours of life.^2^ Neonatal mortality rate remains a challenge; the risk factors associated with neonatal mortality are considered quality indicators for improving health care provided in the Neonatal Intensive Care Unit (NICU), as well as an indicator of population health and wellbeing.^2^

The NICU must have highly sophisticated facilities and equipment to address critical cases, facilitate adjustment of the newborn to the extra-uterine life, and establish and maintain normal respiration of a high-risk newborn.^3^ Although NICU helps to reduce preterm mortality, it is scarce and is a financial burden on the healthcare system in developing countries.^4^

A remarkable decline in mortality rates during neonatal period for the past two decades is due to the advances of obstetric practice in term of medical screening and surveillance, and increased neonatal specialization. However, respiratory tract disorders, along with sepsis and other types of infection, are the major causes of neonatal morbidities and mortalities.^2^ Consequently, the length of hospital stay, intensive care costs, and burden on the healthcare system have increased.^5^

There is a paucity of studies that address neonatal morbidity and mortality as NICU admission patterns vary based on region and time.^6^ However, such studies are necessary to improve the standard and quality of care, which in turn, would facilitate proper resource utilization and enable comparison for health outcomes.^7^ Therefore, this research was aimed to discover patterns and end results of disease and the factors dominating neonatal deaths in the NICU.

## METHODS

This one-year retrospective study (hospital-based) was conducted in the NICU of Princess Rahma Pediatric Teaching Hospital, Irbid; Jordan from 1^st^ of March, 2018 to 28^th^ of February, 2019. This study was approved by the ethical committee of the hospital administration (IRB # 18-02-3135).

The hospital is considered as the main public pediatric teaching hospital in northern Jordan. The catchment area of the hospital is about two million persons. The NICU affiliated to the hospital is a referral unit in the region, and has a capacity of 32 incubators. The NICU has the following facilities: phototherapy, parenteral nutrition, exchange transfusion, artificial respirator, monitoring of carbon dioxide and oxygen level in the blood, oxygen gas supply system, bubble CPAP delivery system, and laboratory tests.

Records of all admitted neonates were reviewed with respect to their medical information. The details were collected using a pretested, structured questionnaire prepared in English. The measured variable (survive or death) was the outcome. While gender of the neonate, gestational age, birth weight, Apgar score, place of delivery, mode of delivery, and length of hospital stay were the controlled variables. The cause of admission and the status at discharge were recorded based on the final diagnosis.

The inclusion criteria of the study were the in-born and out-born neonates admitted to the NICU and diagnosed clinically. Neonates whose data were incomplete, who had ≥ two diagnoses, who were kept under observation, who were taken out of the hospital against medical advice, and who were referred to other hospitals for further medical interventions were excluded from the study. Training was provided regarding data collection techniques to familiarize the data collectors with the instrument.

### Statistical Analysis

The data was examined using statistical software, i.e., SPSS (IBM Corp, IBM SPSS Statistics for Windows, Version 23.0, IBM Corp., USA). To evaluate the demographic data such as age, gender, race and ethnicity, descriptive statistics were used. The proportionate mortality rate (PMR) was calculated by dividing the proportion of cause-specific deaths by the number of deaths from all causes and was then multiplied by 100. The logistic regression was calculated to predict the association between characteristics of newborn at admission and the outcome at discharge. The crude odds ratio was scrutinized with a 95% confidence interval (CI), A p-value <0.05 was perceived to be statistically significant.

## RESULTS

Overall, 1247 neonates were admitted to the NICU during the study period; of these, 703 (56.4%) were male and 544 (43.6%) were female. The majority (n = 1096; 87.9%) were inborn (PRTH) and 151 (12.1%) were referred from other hospitals that have a low level of NICU care. Among those admitted, 776 (62.2%) were full term with the gestational age of ≥37 weeks; the remaining 471 (37.8%) were preterm with the gestational age <37 weeks. Most of the full-term neonates 576 (74.2%) had a normal weight (≥ 2500g) at time of admission, followed by 162 (20.9%) with low birth weight (1500–2499g), and remaining 38 (4.9%) were with very low birth weight (<1500). While in pre-term neonates, 247 (52.4%) were with low birth weight, 167 (35.5%) had a normal weight, and the remaining 57 (12.1%) had a very low birth weight. Regarding the Apgar score at 5 minute, most of the admitted full term neonates (n=731; 94.2%) scored 8-10 (good vitality), followed by those (n=36;4.6%) who scored 4-7 (mild vitality) and the remaining (n=9;1.1%) scored 0-3 (poor vitality). While the Apgar score of preterm neonates most of them (n=275; 58.5%) scored 4-7 (mild vitality), followed by (n= 163;34.7%) scored 8-10 (good vitality), and the remaining (n=32;6.8%) scored 0-3(poor vitality).

The majority of the admitted neonates were delivered via caesarian-section (n = 783; 62.8%), the remaining were delivered normally (n = 464; 37.2%). The average length of hospital stay was 9.6 days; the majority of the admitted neonates stayed in the hospital for ≤7 days (n=783; 62.8%), and the remaining stayed for >7 days (n = 504; 40.4%) (Table-I).

**Table-I:** Descriptive characteristics of the neonates admitted to the NICU (N=1247).

Characteristics	N	%
***Gender***		
Male	703	56.4
Female	544	43.6
***Place of delivery***		
Inborn (PRH)	1096	87.9
Outborn (Referred from outside)	151	12.1
***Gestational age, weeks***		
<33	253	20.3
33–36	218	17.5
≥37	776	62.2
***Birth weight/g (full-term, n=776)***		
<1500	38	4.9
1500–2499	162	20.9
≥2500	576	74.2
***Birth weight/g (pre-term (n=471)***		
<1500	57	12.1
1500-2499	247	52.4
≥ 2500	167	35.5S
***Apgar score at 5 minutes (full-term, n=776)***		
8–10 (Good vitality)	731	94.2
4–7 (Mild vitality)	36	4.6
0–3 (Poor vitality)	9	1.1
*** Apgar score at 5 minutes (pre-term, n=471)***		
8–10 (Good vitality)	163	34.7
4–7 (Mild vitality)	275	58.5
0–3 (Poor vitality)	32	6.8
***Mode of delivery***		
Normal	464	37.2
Caesarian-section	783	62.8
***Length of hospital stay, days***		
≤7	743	59.6
>7	504	40.4

The most common causes of the NICU admission were neonatal sepsis (n = 341; 27.3%), respiratory distress syndrome (RDS; n = 310; 24.9%), and birth asphyxia (n = 163; 13.1%). For sepsis cases, the most common isolated pathogen was Enterobacter 34 (40.5%), followed by Coagulase-Negative staphylococcus 20 (23.8%). The details are presented in Table-II.

**Table-II:** Morbidity profile of the admitted neonates (N=1247).

Reason of admission (final medical diagnosis)	No	%
Respiratory distress syndrome	310	24.9
Respiratory problems	81	6.5
Sepsis/infection	341	27.3
Neonatal jaundice	133	10.7
Meconium aspiration	71	5.7
Birth asphyxia	163	13.1
Congenital anomalies (related to heart, central nervous system and chromosome)	48	3.8
Intrauterine growth restriction	53	4.3
Hypothermia	14	1.1
Infant of Diabetic Mother	23	1.8
Others	10	0.8

Overall, 1147 (91.9%) neonates were discharged after improvement. However, the remaining 101 (8.1%) died. The major causes for mortality according to the PMR analysis were RDS (35.6%), followed by neonatal sepsis (27.7%) and neonatal asphyxia (24.8%). The details are present in Table-III.

**Table-III:** Distribution of admitted cases according their outcome at the time of discharge (N=1247).

Variable (N)	Survival rate n (%)	Death rate n (%)	Total n (%)	Proportionate mortality rate
Sepsis	313 (91.8)	28 (8.2)	341 (27.3)	27.7
Respiratory distress syndrome	274 (88.4)	36 (11.6)	310 (24.9)	35.6
Respiratory problems /TTN	80 (98.8)	1 (1.2)	81 (6.5)	0.99
Birth asphyxia	138 (84.7)	25 (15.3)	163 (13.1)	24.8
Meconium aspiration	69 (97.2)	2 (2.8)	71 (5.7)	2.00
Jaundice	132 (99.2)	1 (0.6)	133 (10.7)	0.99
Congenital anomalies (related to heart, central nervous system and chromosome)	41 (85.4)	7 (14.6)	48 (3.8)	6.90
Intrauterine growth syndrome	51 (98.1)	1 (1.9)	52 (4.2)	0.99
Others	48 (100)	0 (0)	48 (3.8)	0

Total	1146 (91.9%)	101 (8.1%)	1247 (100)	100

The logistic regression model showed that gestational age, weight of the baby at birth and the quick clinical assessment (Apgar) performed within the first five minutes of birth were significantly related with neonatal survival state (*p* <0.05) (Table-IV). Neonates with gestational age <33 weeks were less likely to survive than those with gestational age ≥37 weeks (OR = 0.09; *p* = 0.001). Neonates with birth weight <1500 g were less likely to survive than those with birth weight ≥2500 g (OR = 0.53; *p* = 0.013). Finally, neonates with the Apgar score at 5 minutes of 0–3 (poor vitality) were less likely to survive than those with the score of 8–10 (good vitality) (OR = 0.34; *p* = 0.001).

**Table-IV:** Stepwise multiple logistic regression analysis to identify the predictors neonatal mortality (N=1247).

Characteristics (n)	Survived		OR (95%CI)	p-value

	Yes (1146)	No (101)		
***Gestational age, weeks***			
≥37 (776)	767 (98.8)	9 (1.2)	1	
33–36 (218)	201 (92.2)	17 (7.8)	0.91 (0.76–1.73)	p = 0.624
<33 (253)	178 (70.1)	75 (29.6)	0.09 (0.11–0.29)	p = 0.001
***Birth weight, gm***			
≥2500 (743)	715 (96.3)	28 (3.8)	1	
1500–2499 (409)	384 (93.9)	25 (6.1)	1.03 (0.089–2.3)	p = 0.432
<1500 (95)	47 (49.5)	48 (50.5)	0.53 (1.14–2.39)	p = 0.013
***Apgar score at 5minute***			
8–10 (Good vitality) (510)	504 (98.8)	6 (1.2)	1	
4–7 (Mild vitality) (607)	592 (97.5)	15 (2.5)	0.92 (0.75–3.34)	p = 0. 314
0–3 (Poor vitality) (130)	50 (38.5)	80 (61.5)	0.34 (1.03–3.31)	p = 0.001
***Place of delivery***			
Inborn (1096)	1031 (94.1)	65 (5.9)	1	
Outborn (151)	115 (76.2)	36 (23.8)	0.92 (0.68–3.72)	p = 0.172
***Mode of delivery***			
Normal (464)	420 (90.5)	44 (9.5)	1	
Cesarean section (783)	726 (92.7)	57 (7.3)	1.02(0.91–4.87)	p = 0.079

## DISCUSSION

This research was carried out to describe the factors which decisively affects the morbidity pattern, risk factors and undesirable outcomes, leading to neonatal deaths at NICU, a tertiary care public teaching hospital. In this study, the vast majority of admitted neonates (87.9%) were in-born and the rest were out-born. The outborns were referred from other public hospitals of north of Jordan because the respective NICU were fully occupied, or the neonates required a high level of intensive care. The high percentage of inborns indicates improved awareness of parents regarding birth in the hospital, and is consistent with the Jordan Ministry of Health strategies to reduce the neonatal mortality rate in Jordan.

In the present study, the preterm neonates (37.8%) had a gestational age < 37 weeks at admissions, and the remaining 62.2% had full-term. Similar findings were reported in a study conducted in Ethiopia^8^ in which 34.9% were preterm neonates and 63.6% were full-term neonates. This high percentage of preterm neonates may be due to some risk factors such as rupture of fetal membrane, multiple pregnancy, intrauterine growth restrictions, socioeconomic status, and lack of maternal antenatal care.^9^

Regarding the birth weight, most of the full term neonates 576 (74.2%) weighted ≥ 2500 g at time of admission, and the remaining 200 (25.8%) were weighted ≤ 2500 g. While the majority of preterm neonates 304 (64.5%) were weighted ≤ 2500. The percentage of full term with ≤ 2500g (25.8%) is comparable with studies from Pakistan (37.7%),^10^ and Ethiopia (35%).^8^ In spite of the fact that there are several factors related with low birth weight, the major endangerment is inadequate nutrition during pregnancy, anemia, lack of folic acid supplements, and low socioeconomic status.^11^

Most of the admitted neonates were delivered by caesarian-section (62.8%). Similarly, in a study conducted in Iran by Fallahi et al.,^12^ caesarian section deliveries accounted for 58.6% of the admitted neonates. A study has revealed the advantages associated with cesarean section, including higher 1-minute score, less intraventricular hemorrhage, and lower mortality rate;^12^ However, recently, the prevalence of caesarian-section has increased,^13^ as an elective mode of delivery without an accepted “medical indication” for avoiding labor pain, and to avoid mothers some embarrassing situation during vaginal delivery, such as urinal incontinence and fecal defection.^14^

In this study the average length of hospital stay was 9.6 days, and the majority stayed for ≤ seven days. An identical study was carried out in Nepal where the majority of admitted neonates stayed for 6-10 days.^15^

The most common causes of admission to the NICU were neonatal sepsis (27.3%), followed by RDS (24.9%) and asphyxia (13.1%). This finding is more or less the same as it was observed in the study conducted by Kanodia et al.^15^ Sepsis and perinatal asphyxia were also found to be the cause for admission to the NICU in a study conducted by Gaucham et al.^16^ However, a study conducted in Pakistan, neonatal sepsis was found to be as low as 2% lower than the rate shown in this study.^17^ This discrepancy may be due to the different diagnostic approach (diagnosed clinically or by lab culture test). RDS was the most common cause of neonatal morbidity in preterm neonate in other studies.^18^ The order of these diseases as the common causes of admission varies from one study to another depending on the risk factors and the criteria used to diagnosis the diseases.^19^

The predominantly isolated pathogen in the study was Enterobacter, the other pathogens such as Coagulase–Negative Staphylococci, and Klebsiella were less common. A study on the Pakistani population, Escherechia Coli was the most common organism followed by Klebsiella, and among the gram-positive organisms, Staphylococcus Aurous was most frequent.^20^

The death rate observed in our study (8.1%) is similar to the death rate observed in a research conducted by Manktelow et al.,^21^ and was lower in a study carried out by Rakholia et al.^22^ Respiratory distress syndrome (RDS), neonatal asphyxia resulting from deprivation of oxygen and sepsis were the three most usual causes of mortality. This may be due to poor antenatal care, maternal risk factors, and delay in referral from peripheral hospitals.^23^

Logistic regression model predicts that gestational age, weight of the baby at birth, and Apgar score performed within the first five minutes of birth neonatal mortality. A study conducted in Ethiopia has the same findings.^24^ This could be explained by the fact that prematurity increases the likelihood of underweight, and increases the risk complications such as sepsis, other infections, and birth asphyxia.^25^

### Limitation of the study

This research was performed by studying the available records at hospital and could not address some more topics because of limitations. As it was a clinical based study that examined data from the medical records of neonates in a single health facility, the findings cannot be generalized.

## CONCLUSION

This study shows that neonatal sepsis, neonatal RDS, and perinatal asphyxia are leading grounds of morbidities in newborns, and are responsible for a high rate of mortality but the order is different. Understanding the cause of morbidity and mortality of neonates admitted at NICU is useful for prioritizing and planning health services, re-allocating resources, and improving the quality of care.
